# Sensory Descriptor Analysis of Whisky Lexicons through the Use of Deep Learning

**DOI:** 10.3390/foods10071633

**Published:** 2021-07-14

**Authors:** Chreston Miller, Leah Hamilton, Jacob Lahne

**Affiliations:** 1 Data Services, University Libraries, Virginia Tech, 560 Drillfield Dr., Blacksburg, VA 24061, USA; 2 Department of Food Science & Technology, Virginia Tech, Blacksburg, VA 24061, USA; hleah@vt.edu (L.H.); jlahne@vt.edu (J.L.)

**Keywords:** natural language processing, deep learning, sensory science, flavor lexicon, long short-term memory

## Abstract

This paper is concerned with extracting relevant terms from a text corpus on whisk(e)y. “Relevant” terms are usually contextually defined in their domain of use. Arguably, every domain has a specialized vocabulary used for describing things. For example, the field of Sensory Science, a sub-field of Food Science, investigates human responses to food products and differentiates “descriptive” terms for flavors from “ordinary”, non-descriptive language. Within the field, descriptors are generated through Descriptive Analysis, a method wherein a human panel of experts tastes multiple food products and defines descriptors. This process is both time-consuming and expensive. However, one could leverage existing data to identify and build a flavor language automatically. For example, there are thousands of professional and semi-professional reviews of whisk(e)y published on the internet, providing abundant descriptors interspersed with non-descriptive language. The aim, then, is to be able to automatically identify descriptive terms in unstructured reviews for later use in product flavor characterization. We created two systems to perform this task. The first is an interactive visual tool that can be used to tag examples of descriptive terms from thousands of whisky reviews. This creates a training dataset that we use to perform transfer learning using GloVe word embeddings and a Long Short-Term Memory deep learning model architecture. The result is a model that can accurately identify descriptors within a corpus of whisky review texts with a train/test accuracy of 99% and precision, recall, and F1-scores of 0.99. We tested for overfitting by comparing the training and validation loss for divergence. Our results show that the language structure for descriptive terms can be programmatically learned.

## 1. Introduction

### 1.1. Flavor Language

Flavor is a major factor motivating eating behavior and food choice, but due to the approximately 350 different receptors for aroma-active compounds and the low detection thresholds for many such compounds [1], it is not currently possible to predict a flavor experience from chemical data alone. On the other hand, English and most other languages do not have a systematic and unambiguous flavor vocabulary, so studying flavor by surveying humans is still challenging. Sensory scientists need a way of aligning the different sensory lexicons used by different tasters and stakeholders.

The earliest solution in Sensory Science was the Descriptive Analysis (DA) panel, a body of related methods that use the experiences and vocabularies of a small panel of participants to create a single aligned sensory lexicon for some category of products. In order to ensure alignment between panelists (namely preventing needless synonyms and disagreement about definitions), every word is defined in reference to a physical standard. The largest time investment (often taking weeks or months) in DA is the hands-on training to identify appropriate descriptors and references, then creating experts in the newly-defined language, all of which occurs before the product analysis [2].

This standardized vocabulary is called a sensory or descriptive lexicon and comprises words or phrases called “descriptors”: terms that can be used to describe the flavor, aroma, mouthfeel, taste, appearance, or other sensory attributes of the product set [3]. For highly-studied categories or in cases where flavor communication between groups is important, the lexicon itself may be a desired outcome [3,4]. Lexicons can provide a reference list of possible terms for analysis of products by trained or untrained panelists; lexicons can be used to communicate sensory properties of products to consumers for marketing and product differentiation, and lexicons can be used to define product categories by connoisseurs and enthusiasts, as prototypically documented in the wine world [3,4,5].

Smaller research operations and newer or less-studied categories, however, often prefer methods of flavor measurement that do not need a carefully crafted flavor lexicon. These “rapid” methods collect similarity measurements, allow consumers to use their own, untrained vocabulary to describe product flavors or both [6]. When flavor is described with colloquial language, there are likely to be individual differences and other problems like those encountered during DA training, but rapid methods deal with these problems after data collection rather than before.

The process of identifying meaningful descriptors from free-text product descriptions and combining synonymous terms is known as comment analysis (or free text analysis), and its adoption within Sensory Science has made it possible to utilize existing sources of descriptive text as sensory data. Comment analysis of existing text data has previously been used to produce or modify lexicons for rum [7], wine [8], and whisky [9] and to identify terms that drive liking or price in wine [10,11]. Like all descriptive lexicons, these will never be truly universal—a new product can have a new taste, or a new consumer can have a new perspective on the products—but lexicons are universalizable: they are “intersubjectivity engines” that allow structured communication about the subjective, perceptual qualities of foods, beverages, and other consumer products [5,12,13].

Because it requires human attention, the scale of DA is necessarily limited: the largest DA studies usually include no more than about 100 products. In comparison, there are thousands or even tens of thousands of whiskies currently on the market [14], hundreds of thousands of wines, and similar variety in other specialty food markets, like coffee, chocolate, beer, tea, and so on. All of these products are sold partly or entirely based on the value of their sensory attributes, and these attributes are described in various publications such as reviews and commentaries. While manual comment analysis requires fewer work hours than DA, it is still impractical for large datasets like the RateBeer or Yelp review corpora [15,16]. Computational methods are needed to fully leverage the power of existing sources of food-descriptive data.

### 1.2. Natural Language Processing and Machine Learning

The field of Natural Language Processing (NLP) has quickly matured in the last several years with the boom of deep learning techniques. A number of techniques have been developed in NLP for the identification of relevant terms in freeform text, but accomplishing this task in any given application is usually challenging due to the importance of domain-specific language and unique words not well-represented in language references like thesauruses.

Previous computational linguistic analysis of flavor in food descriptions has relied on having text with a high density of flavor descriptors [10,11], which is not always the case [8]. There are no published tools designed specifically for the identification of flavor descriptors. In this paper, we will prototype and test such a tool using a Long Short-Term Memory (LSTM) deep neural network [17] trained on manually-annotated whisky reviews. The architecture used is based off of a keyword extraction tool developed for identifying skills in resumes [18].

We believe that the flexibility of the Long Short-Term Memory (LSTM) deep neural network architecture in capturing context in natural language will allow the identification of unique language lexicons from whisky reviews. Free comments (reviews) are the most natural way for humans to describe their food experiences, but they are very hard to systematically analyze, especially in volume. The use of LSTMs presents an alternative to hand-coding and increases the volume of data we can meaningfully deal with. The ability to take advantage of previously written reviews to build lexicons with minimal human intervention has much value. Our investigation is a use-case scenario for LSTMs, but not the only one. This kind of architecture (and related ones) can probably solve many problems in Sensory Science (including sentiment and synonymy/similarity) as Sensory Science has seen very limited use of NLP.

### 1.3. Objectives

Our overarching aim is to be able to use preexisting reviews to create a flavor language by identifying and extracting the unique descriptors. Since the world of food and beverage descriptions is obviously a large and heterogeneous domain, we use whisk(e)y as a case study for this domain, as it is an important economic product [14] and one without an authoritative flavor language that comprehensively covers the many increasingly-relevant product styles [19]. This approach should, in principle, be applicable to any product with a large corpus of free-text description available, such as the flavor of other foods, textile feel, and perfume aroma.

To determine whether it is possible to separate flavor-descriptive terms from those with no sensory meaning in written descriptions of food experiences, such as those found on product review blogs, we created a data pipeline that uses NLP techniques to take freeform text whisky reviews and extract descriptors, which can be used to create a lexicon for each whisky: a list of characteristic descriptors or a descriptive representation. The results can be used to identify relationships between descriptors and allow us to begin understanding the flavor language of whiskies.

Therefore, our contributions are threefold:

- An interactive annotation tool to facilitate identifying descriptors and non-descriptors, which allows the creation of a training set for a deep learning architecture;
- The deep learning architecture for our problem domain; and
- The pipeline for data preparation, annotation, training, and testing not seen before in Sensory Science.

## 2. Materials and Methods

The training of a neural network requires a training set of positive and negative examples—in this case, words that are descriptive of flavors as opposed to other words in product reviews that do not describe flavor. To minimize the burden of manual annotation, we developed an interactive visual tagging tool. Full-text reviews were preprocessed to provide a list of potentially descriptive word forms for human annotation as descriptive or non-descriptive, and then individual instances of these descriptive and non-descriptive words in context were used to train and test the proposed LSTM architecture.

### 2.1. Data Collection

The dataset we used to train our model contains a total of 8036 full-text English whisky reviews scraped from four websites: WhiskyAdvocate (WA; 4288 reviews), WhiskyCast (WC; 2309 reviews), The Whiskey Jug (WJ; 1095 reviews), and Breaking Bourbon (BB; 344 reviews). WA and WC reviews are from websites affiliated with a magazine and podcast, respectively, and written by professional reviewers scoring whiskies from around the world and providing tasting notes. BB and WJ are smaller “semi-professional” review websites more focused on American whiskey. BB and WA have multiple named reviewers writing the tasting notes for their websites, while WC and WJ each have a single named reviewer.

WC, WJ, and BB were scraped using beautifulsoup4 in Python v3.7, while WA was scraped using the rvest package v0.3.2 in the R Statistical Environment v3.5.3. A combination of GET-specific scrapers were used to collect the review-containing URLs from the various sites, and then the content was collected with site-specific scrapers. When possible, page formatting was used to collect metadata about the products being reviewed such as country of production or the proportion of alcohol by volume (ABV), as well as the review itself, but the metadata were not consistent across sites.

### 2.2. Data Preparation

After collection, each review was converted from full text (excluding the title and metadata elements such as the date of publication) into a list of potentially-descriptive word base forms called “lemmas” that occurred in each review using the workflow described in [19]. Briefly, the reviews were first tokenized, or converted into an ordered list of individual words and punctuation. Each token was tagged with a part-of-speech (POS) label such as “adjective” or “punctuation”, and all tokens other than nouns, adjectives, and a small whitelist of verbs were removed. The remaining tokens were lemmatized, or converted into their base form (e.g., “drying“ to “dry”).

This was done using Spacy v2.1.8 in Python v3.7. The pretrained model en_core_web_sm v2.1.0 was used as the basis for calculating predictions and R package cleanNLP v3.0 was used in R v3.5.3 to convert the data to a tabular CSV format.

These lemmas were used as the list of potential descriptors for manual annotation with our interactive visual tagging tool (described in the next section). The frequency of occurrence (as an adjective or noun) for each lemma was used to prioritize more common lemmas for annotation.

### 2.3. Interactive Tagging Tool

**Figure 1:**
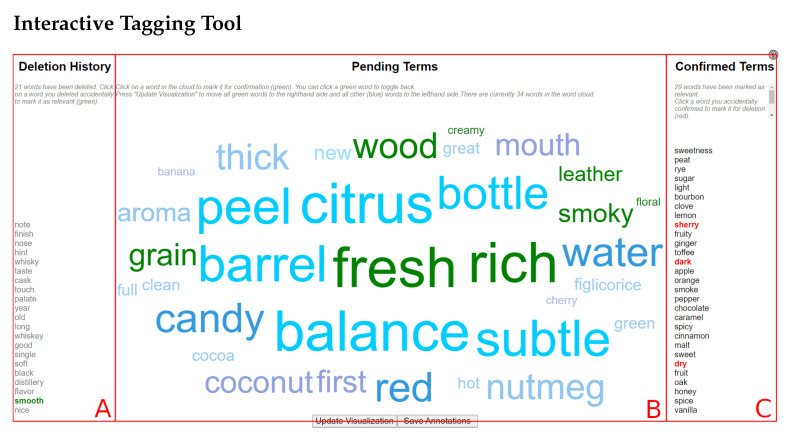
Interface for the Interactive Tagging Tool: (**A**) Non-descriptors (negative examples) are kept in the deletion history. (**B**) Then the most frequent words (up to 50) are shown in a word cloud format. (**C**) Confirmed descriptors (ones that are selected by the human operator) are stored in a confirmed terms list.

To create examples of descriptive and non-descriptive words in context for this study, human annotators used a browser-based interactive tagger tool based on a word cloud visualization, seen in Figure 1. The tool was built for this purpose in Javascript and HTML5 using jQuery v3.4.1. A CSV file of token frequencies is uploaded from the user’s local storage, the most common terms are rendered into a word cloud using jQCloud v2.0.3, and the user assigns words to the descriptor (1) or non-descriptor (0) classes using one of three interaction modes. The central wordcloud display (Figure 1B) repopulates with progressively less common words as the user assigns words to classes, and the resulting corpus of labeled words is exported along with unlabeled words as a CSV file to the user’s local storage. Up to 50 words can be displayed in the central panel at a time, based on the rendering algorithm described in [20]. Fancybox v3.5.7 is used to display tooltips.

With a low learning curve, the user is able to sift through the text in a timely manner and create a human-annotated list of positive and negative examples. The user is then able to save the corpus of labeled words to a comma separated value (CSV) file.

### 2.4. Gold Standard Annotations

We asked four annotators (A, B, C, and D) from Food Science to use the interactive tagger tool to create an annotated training set. The annotators were chosen based on their expertise in Sensory Science, a sub-field of Food Science. Annotators A and B were involved with annotating all the datasets, while C was a tiebreaker for datasets WA and WC and D was a tiebreaker for BB and WJ. A lemma was deemed a descriptor if it was tagged as such by two out of the three annotators; otherwise, the lemma was tagged as not being a descriptor. As such, the number of annotators was chosen so a best two out of three consensus could be achieved. This is important as it provides a more accurate set of labeled annotations and is a common practice in both corpus annotation in NLP [21] and in the analysis of freeform comments in sensory science survey research [22]. A total number of 1794 lemmas (499 descriptive, 1295 non-descriptive) were tagged and used to create a training and test set. There were a total of 2638 unique descriptive/non-descriptive tokens tagged based on these lemmas (e.g., the lemma “fruit” could appear in the text as “fruity” or “fruits”, i.e., a lemma could result in multiple tagged tokens). All individual occurrences of the tokens in context were used for training.

### 2.5. Word Embeddings

A word embedding is a representation of a word as a high-dimensional vector. The closer a pair of word vectors are in the high-dimensional space, the more the words are conceptually “similar” or “related”. An input sequence for each word (i.e., potential descriptor) was created from the context and potential descriptor (unigram). Each potential descriptor and context word is assigned a 300 dimensional GloVe [23] word embedding. GloVe embeddings with 1.9 million tokens were used. A key note is that terms generally used in a domain specific language, such as those of whisky tasting notes, are not commonly used by the lay person, so this is a key consideration for domain-specific keyword extraction.

**Figure 2:**
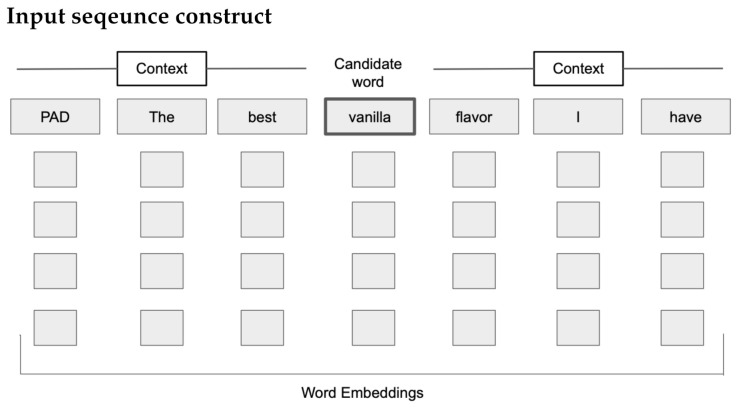
Conceptual example of an input sequence for training the model. The before and after context of a candidate word is extracted from a text review. This is then converted into a numerical representation using GloVe word embeddings for each context word and the candidate word with a PAD being the zero vector.

As illustrated in Figure 2, three words before and after were used as context, n = 3. If the context was less than three words; e.g., if the word was the first word of a sentence, then a PAD, a filler value, was used to signal no available context. The PAD value is assigned the zero vector. It is these input sequences that were used to train the model described in the next section.

### 2.6. Descriptor Extractor

We chose a uni-directional Long Short-Term Memory (LSTM) deep neural network architecture since it works well with the context of language. An LSTM is a Recurrent Neural Network (RNN) designed for modeling sequence data. LSTMs have a memory segment that can “remember” up to a certain degree of events in time. Hence, it works well with remembering context in language and the relationships between words. The context that a descriptor is found is essential to identifying what is or is not a descriptor. How a word is used can be a deciding factor.

**Figure 3:**
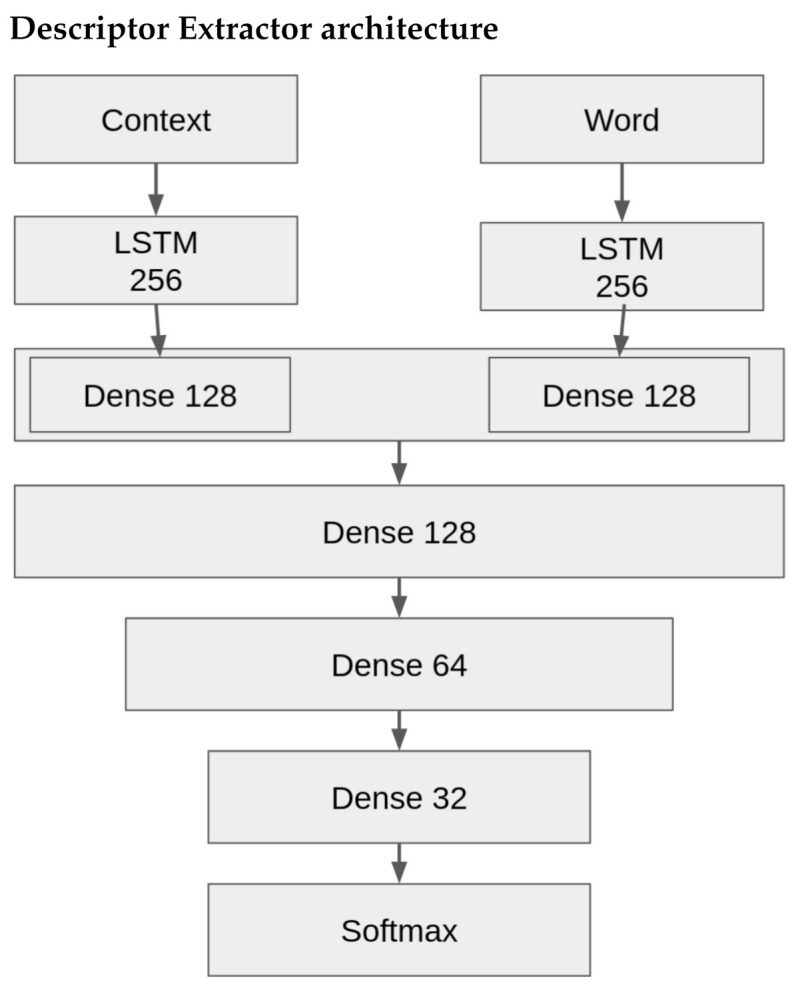
Deep Learning Architecture which is composed of two sets of input (context and a word) that feed into an uni-directional LSTM each. The rest of the architecture concatenates the LSTM layers and continues to merge dense layers with a softmax as the output.

The architecture used was inspired by [18] and can be seen in Figure 3. There are two inputs, the context of a potential descriptor and the potential descriptor itself. Each is fed into a uni-directional LSTM of 256 units followed by a dense layer of size 128 units. These two dense layers are concatenated and fed forward to a series of decreasing dense layers ending with a binary softmax output layer that decides whether the input word is a descriptor or not. Defining the size of the context is flexible while we currently fix the descriptor (word) to a unigram as we observed most descriptors are single words. However, we chose a context of three words before and after a descriptor, n = 3.

We decided to use a traditional LSTM as a starting point for our keyword (descriptor) extractor. We wanted to see how well this model structure could perform before turning to more sophisticated model architectures in the future such as transformers [24]. As we will discuss in our results, the model architecture performed well.

The Descriptor Extractor was written in Python v3.6.9 with the deep learning architecture built using Keras v2.3.1 and Tensorflow v1.14.0. Comet.ml [25] was used to track different aspects of the model training, allowing us to provide detailed information presented in some of the figures in the Results section.

## 3. Results

Our experiments focused on testing our LSTM architecture as it was tailored to the problem space. For a comparison baseline, we chose parts-of-speech (POS) tagging since it closely reflects the characteristics of descriptors, which are generally adjectives and nouns. The POS approach is currently state-of-the-art for Sensory Science and therefore reflects a valid comparison [10,19].

Our first experiment combined the WA and WC datasets into one dataset for training and validation. We used an Adam optimizer with a learning rate of 0.0001, and our loss function was binary cross entropy with a batch size of 32. We had the BB and WJ datasets annotated in the same fashion as WA and WC so as to have a labeled test set. The results on the test set (accuracy/precision/recall/F1-score) can be seen in Table 1. The scores were lower compared to those of the training set. This made us rethink why this could be happening. We realized that an important difference between WA/WC and BB/WJ was that WA/WC were professionally written reviews, whereas BB/WJ were hobbyist reviews. There are likely different writing styles between the two kinds of reviews driving this difference in performance.

We then combined all the datasets (WA, WC, BB, WJ) into one dataset and performed a train/test split of 80/20. We approached our methodology of splitting up the train and test sets differently. After combining WA, WC, BB, and WJ, we tokenized the reviews into words (tokens) and performed a train/test split on the tokens themselves instead of on a review basis. We also recorded the specific review it occurred in, the sentence within the review, and the position in the sentence. Therefore, instead of just performing a search for all locations of vanilla, for example, in all reviews for each training and test sets, we used the specifically tagged location for each instance of vanilla. From there, we were able to extract the context (n = 3 words before and after each token). Hence, we isolated where each instance of vanilla was for the respective training and test set. Combining the reviews to create a new train and test set allowed the model to be exposed to more variations in writing styles and hence become a more robust classifier.

Given the labeled descriptors/non-descriptors (2638 unique), we identified around 250K instances of the labeled words. As mentioned, we used a randomly chosen 80/20 split for training and testing. The total number of words used for training and validation were around 200K for training and around 50K for testing. For training, we removed punctuation but kept stop words as they are part of the context. Twenty percent of the training data were used for validation. Each training/test split contained a class ratio of 56% non-descriptors and 44% descriptors; hence, there was no class imbalance.

**Figure 4:**
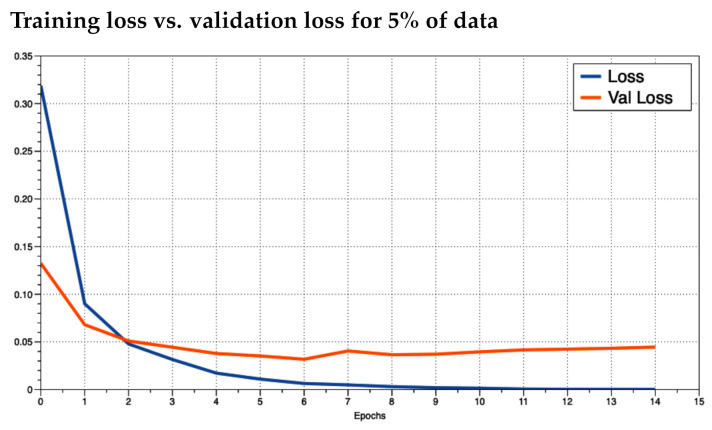
Training Loss versus validation loss using 5% of the training data where the *x*-axis is the epoch and the *y*-axis is the loss.

**Figure 5:**
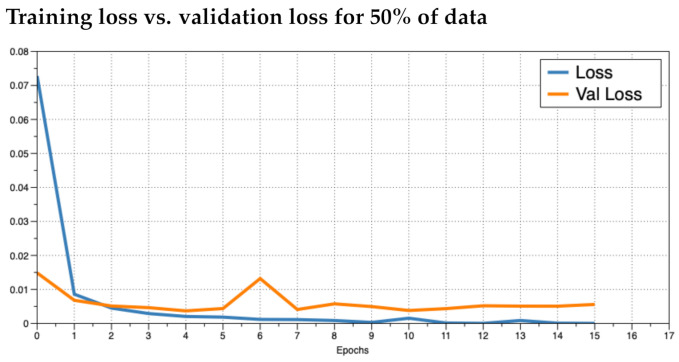
Training Loss versus validation loss for using 50% of the training data where the *x*-axis is the epoch and the *y*-axis is the loss.

**Figure 6:**
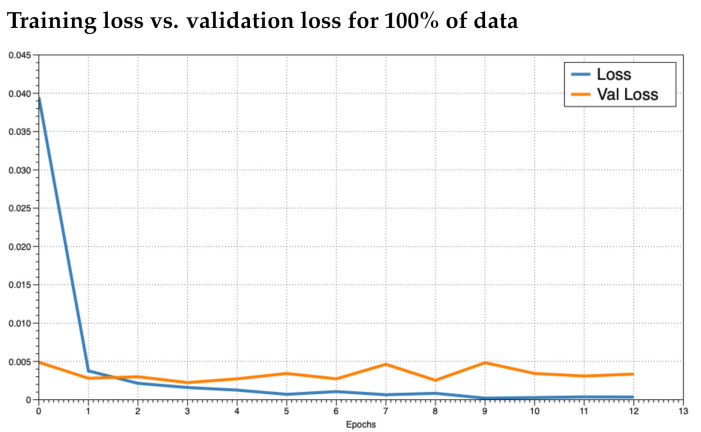
Training Loss versus validation loss for using 100% of the training data where the *x*-axis is the epoch and the *y*-axis is the loss.

It was unclear as to how many epochs to train for. An epoch is the number of passes through the entire training dataset. We noticed that the accuracy converged to near 100% quickly (within the first two epochs). To prevent overfitting, we ran the training for as many epochs as necessary until the loss did not improve and then plotted the training loss versus the validation loss to see how the training was behaving. We trained the model in increments of 5% use of the data up to using 100%. This resulted in 20 training sessions. This was done to observe how the model behaved given different numbers of training data. In practice, if a model performs poorly, the inclusion of more training data may improve results. We investigated the plots for 5%, 50%, and 100% (Figure 4, Figure 5 and Figure 6, respectively). We observed that the loss values consistently crossed roughly around three epochs an then diverged (overfitting). This is marked as “Epoch 2” in the figures as Epoch 1 is really Epoch 0 in the figures. Hence, we chose to train for three epochs.

**Figure 7:**
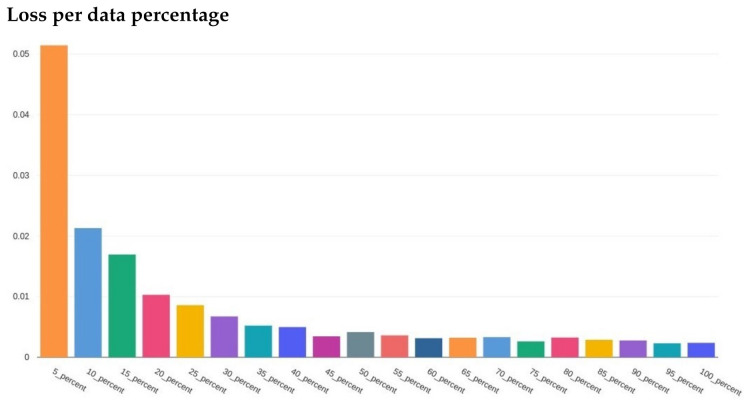
The loss for each percent of the training data used. *x*-axis is the specified percentage used and *y*-axis is the loss value.

**Figure 8:**
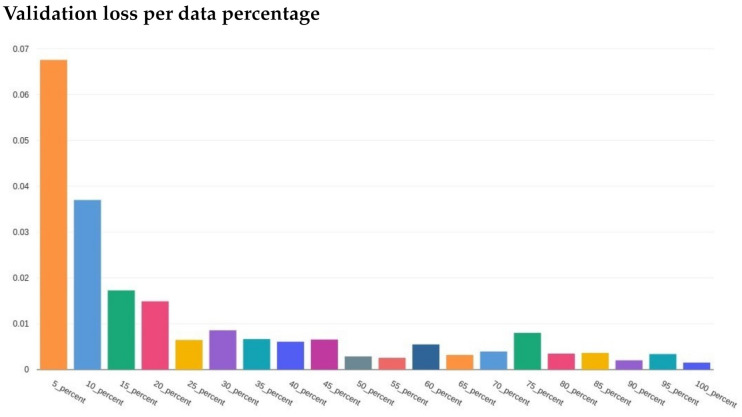
The validation loss for each percent of the training data used. *x*-axis is the specified percentage used and *y*-axis is the loss values.

After reviewing the loss and accuracy for each incremental iteration, we decided to report on using 100% of the training data. The gain from using all the data was small, e.g., loss difference of 0.00231 loss for 95% of the data versus 00.00238 for 100%. The difference in accuracy was equally minimal. Since the training with 100% of the data did not take long (around 5 min for three epochs using a desktop CPU), the small increase was still worth the extra training time. Figure 7 and Figure 8 illustrate the training loss and validation loss, respectively, in which each loss (*y*-axis) is plotted in comparison to the percent of the data used in training (*x*-axis).

**Figure 9:**
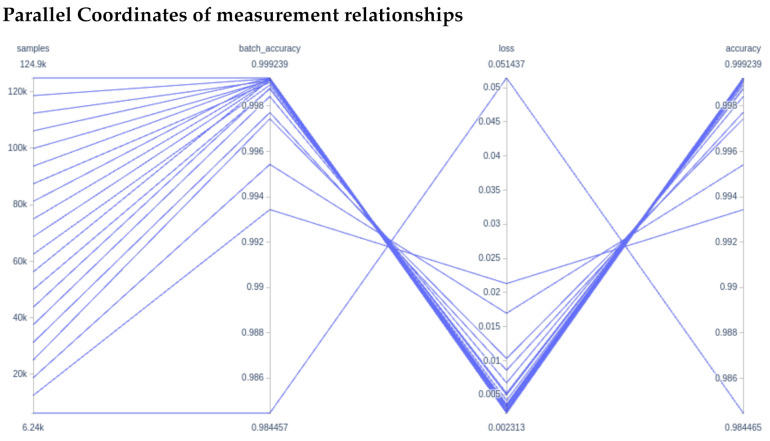
From left to right: The relationships between the number of samples used for training, the batch accuracy, the training loss, and the final accuracy.

We then use a parallel coordinate system (Figure 9) to illustrate how the use of more samples (higher percentage of data used) increases the batch accuracy, which corresponds to a lower loss and an overall higher accuracy. Note that the axis ranges for batch accuracy, loss, and accuracy are quite small.

The results from training can be seen in Table 2. Here the loss and accuracy are reported for the cases where available. Our model’s training accuracy hovered at 99% with POS at 51.3%. The precision, recall, and F1-scores for the test set are presented in Table 3. One thing to note is that the recall is very high for POS. POS classifying is essentially saying “all nouns and adjectives are descriptors”. In that case, there will be very few false negatives, because almost all descriptors ARE nouns and adjectives. Since recall = true positives/(true positives + true negatives), recall will be very high.

**Figure 10:**
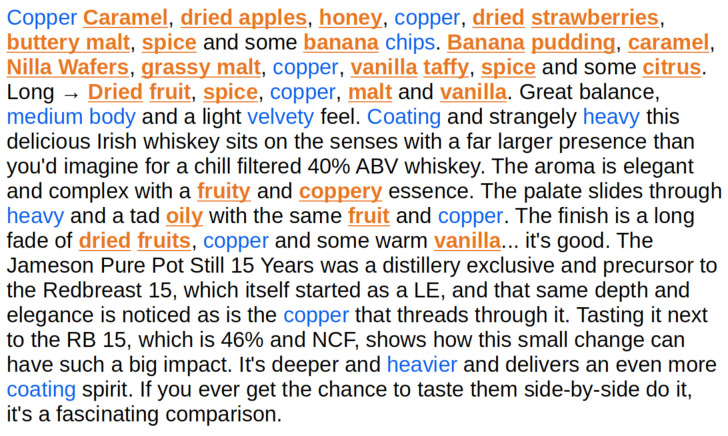
Prediction results for a random review. The orange underlined words were identified by the LSTM model. The blue ones were not identified.

An illustration of a case where the LSTM model struggles can be seen in Figure 10. The orange underlined words were identified by both a human annotator and the LSTM model. The blue ones were not identified by the LSTM model but were by the human annotator. The primary differences tend to be that the LSTM model only identifies the more descriptive word in bi-gram descriptor phrases (e.g., “banana chips“) and will classify uncommon words, especially proper nouns, as descriptive, albeit with a low probability (e.g., “Redbreast”, prediction of 71%). The challenge with bi-grams is a focus of future work discussed later, and the low probabilities can be addressed by using a filter threshold.

Figure 10 also demonstrates the difficulty in creating a tagged gold standard corpus, as words like “copper” and “heavy” that are not usually flavor words were annotated by the LSTM model as non-descriptors. In certain contexts, as in the idiosyncratic text of this review, these words are arguably capable of describing flavor. In the majority of reviews, however, “copper” instead describes the color (not flavor) of the spirit. The difficulty that these kinds of rarely descriptive words present for rapid annotation is also a focus of future work for the tagging scheme described in this paper.

We were also interested in viewing the relationships between words chosen as descriptors or non-descriptors. One approach to do this is to visualize the GloVe word embeddings using a t-SNE plot, an approach to visualize high-dimensional data in a two-dimensional space [26]. What results is a scatter plot visualization where distance between each word represents “similarity” based on word embeddings. The closer they are, the more conceptually similar.

**Figure 11:**
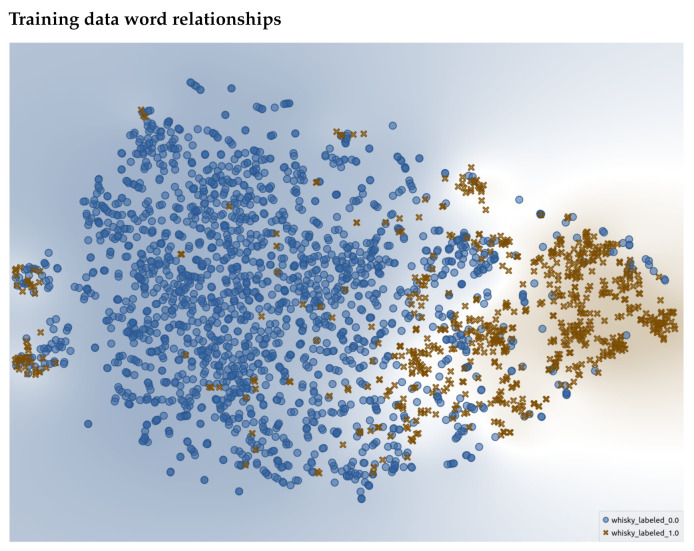
t-SNE representation of the training data where a blue dot represents a word labeled as a non-descriptor, and a brown ’‘X” represents those that are descriptors.

**Figure 12:**
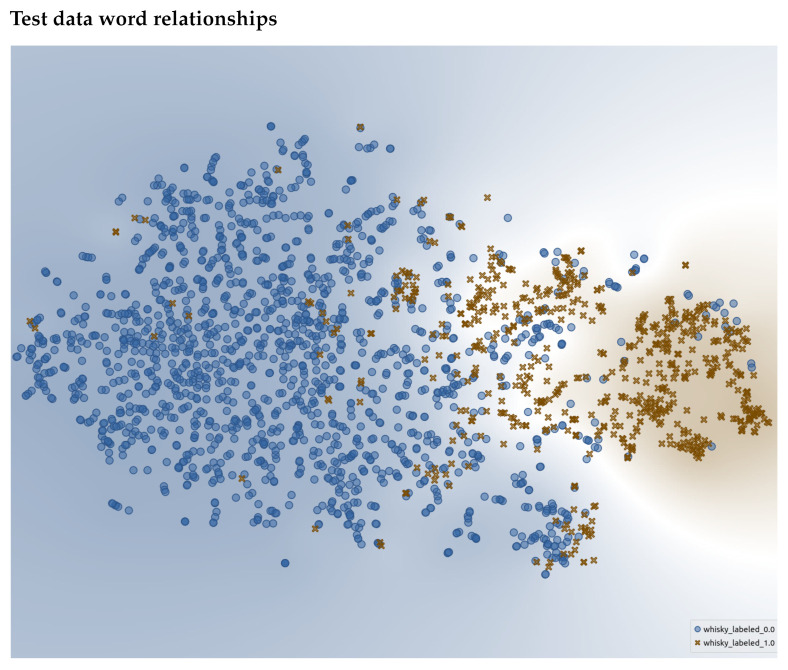
t-SNE representation of the test data where a blue dot represents a word labeled as a non-descriptor and a brown “X” represents those that are descriptors.

**Figure 13:**
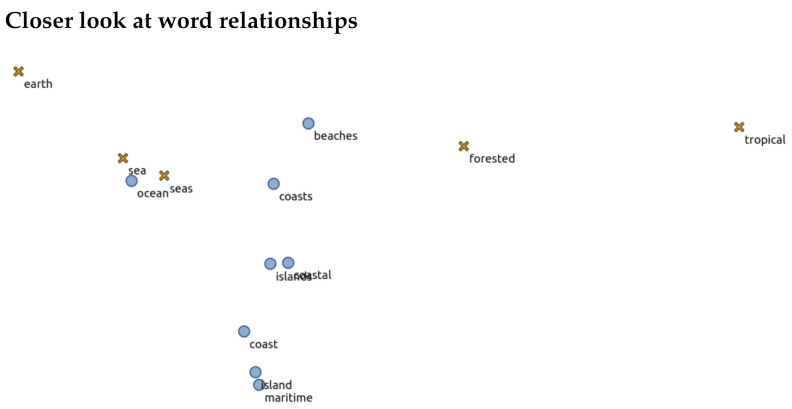
A zoom-in of a t-SNE plot to exemplify the separation of words into sensory and non-sensory despite their similarity of word embeddings. This shows that some words can hold a form of similarity but are not deemed “equal” in a descriptive sensory sense.

We first plot the t-SNE for the annotated words within the training set. This can be seen in Figure 11 with a descriptor being a brown “X” and non-descriptor a blue dot. The words that were labeled in the training dataset create distinct clusters. This demonstrates that the human annotations created a well-defined cluster space, and hence, supports that the provided annotations were of good quality and the embeddings have enough understanding of flavor language to have captured it in the embedding space. The same can be said for the clusters for the test dataset (Figure 12). One may notice that there are some descriptor/non-descriptors “speckled” across the opposing cluster, i.e., words labeled as a descriptor are found within the non-descriptor cluster. This demonstrates that just because words are similar in an embedding space, their contextual meaning can vary. Zooming in to an example (Figure 13) of this, we see various terms that describe different aspects of bodies of water or climates. These words hold some form of similarities but are not deemed “equal” in a descriptive sensory sense.

**Figure 14:**
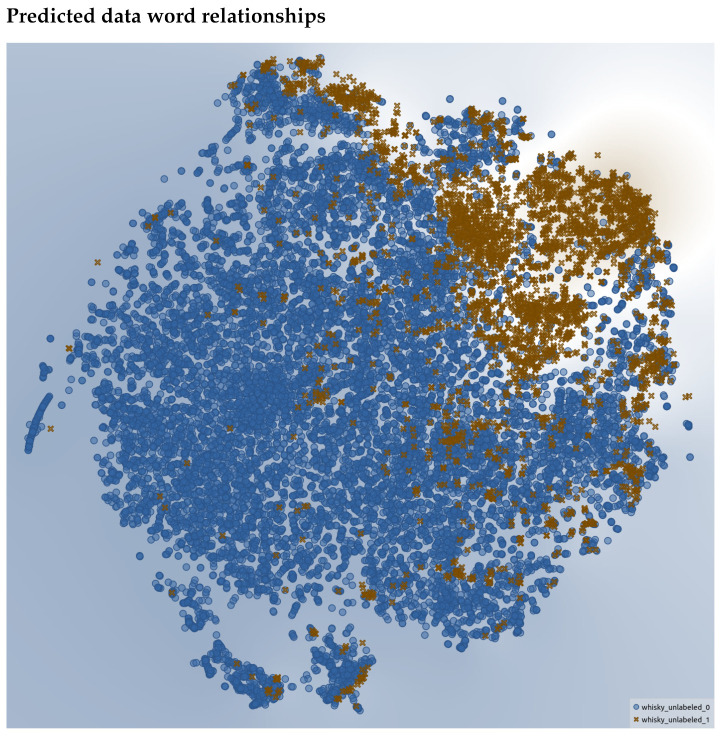
t-SNE representation of the words that were not annotated. A blue dot represents a word predicted as a non-descriptor, and a brown “X” represents those that are predicted as descriptors.

In Figure 14, we see the embedding space for words that were predicted to be a descriptor (brown “X”) or not a descriptor (blue dot). As can be seen, neatly defined clusters also emerge along with some “speckles”. This provides support that the trained model is able to segment the space into descriptors and non-descriptors and hence carve out sensory terms. Another observation is that in all the t-SNE embedding plots, the non-descriptors outnumber the descriptors as was noted to be the case in the Related Works section by [8]. The actual descriptors are the minority, which makes sense as we observed that in the reviews, most words are non-sensory.

## 4. Discussion

We were able to build a deep learning model architecture based on LSTMs to provide descriptor identification within free-form text whisky reviews. Our results were very promising with training, validation, and test accuracies around 99%. The precision, recall, and F1-Scores were equally high. This is substantially higher than the current state of the art for Sensory Science. We were concerned about overfitting with such high scores, so we tracked the training and validation loss over many epochs, a common approach to detect overfitting. The tracking showed overfitting after three epochs, so we stopped our training at three epochs.

We were successfully able to automatically separate flavor-descriptive terms from those with no sensory meaning in written descriptions of food experiences (reviews). Our LSTM architecture was able to capture the language constructs that dictate what is and is not a descriptor. We view this as one of our key contributions.

Another key contribution is the pipeline for data preparation, annotation, training, and testing not seen before in sensory science. This opens the door for researchers in Sensory Science and Food Science in general.

We also introduced a novel interactive word tagging tool for creating a set of human-labeled descriptor/non-descriptor words. With multiple annotators using the tool, the set of human-labeled words provided an excellent training set. This supports the possibility of performing the same annotation with other datasets in other domains in order to facilitate the creation of a labeled set of words, hence training a model for those domains.

Visualizing the results using t-SNE revealed some interesting results. First, the embedding space of human labeled words by the interactive word tagger was segmented fairly cleanly into two clusters: those words that are descriptors and those that are not. This supports that the annotations are of high quality. Similarly, the embedding space for predicted words from the test set (non-annotated words) reveals two fairly clean clusters for descriptors and non-descriptors. This provides support that the trained model is able to learn the language structure of sensory terms.

These contributions result in some interesting and novel implications. We trained a model that can identify descriptors in texts, leading to the ability to create a lexicon for a whisky. Lexicons allow a comparison between whiskies: a $50 bottle of whisky can have a similar lexicon to that of a $300 bottle. This allows the consumer to “experience” the $300 whisky by trying the $50 whisky. Lexicons of whiskies can also map distinct descriptors to that of different metadata of the whiskies, such as age, region of origin, ingredients, and price. This can provide a foundation to perform predictive analyses, such as Random Forests. By fitting a model to predict continuous variables (e.g., bottle price, quality score) or classify products (e.g., region of origin) based on the presence or absence of flavor terms in the bodies of reviews, we can identify which flavors or flavor terms drive the price and consumer liking of whiskies or differentiate between product categories.

## 5. Limitations and Advantages

While our approach has had much success, there are a few limitations. The interactive tagger currently does not present the context of a word when the word is shown to the user. The context can influence whether some words are descriptive or not (e.g., “maple” in “a sweet maple aftertaste” vs. “this new offering from Maple Leaf Spirits”). Furthermore, unigrams are used to train the model with a context window of n = 3. Although this context window is based on the suggestion from [18], experimentation with other windows could be beneficial for our domain. The use of unigrams is also a limitation as some descriptors are not unigrams but phrases of two or more words. Studying beyond unigrams would be an important direction for future work.

The LSTM developed in this work is most immediately applicable to situations where researchers have some structured data about products (e.g., price, hedonic liking scores, chemical data, ingredient concentrations) and freeform product descriptions but no structured descriptive data. The presence or absence of the descriptors in each product, or the number of participants who used them, can be used very similarly to check-all-that-apply data in sensory analysis [10,27,28]. Freeform textual data is easier to collect than trained descriptive panel measurements, meaning that the use of this tool could reduce the barrier to entry for studies looking to relate production variables to resulting sensory properties or sensory properties to product liking and consumer behavior. The performance of the LSTM on descriptions of other foods (e.g., other spirits, wine, coffee, specialty meats and cheeses, casserole recipes) should be assessed before using the predicted descriptors for this kind of further analysis, but the interactive annotation tool developed in this work should reduce the amount of work necessary to tag small test sets and, if necessary, new training sets for these other domains.

## 6. Conclusions

In conclusion, we were successful in automatically detecting words as flavor-descriptive terms and separating them from non-sensory terms. Our developed deep learning architecture proved successful and opens the doors for further research into descriptive analysis.

For future work, we would like to perform testing of generalization between food domains, e.g., apply the model to cocktail and coffee descriptions. Currently, we use only unigram descriptors/non-descriptors. We would like to expand to using bi-grams and tri-grams as some sensory descriptors are phrases and not single terms (e.g., “wet dog”, “red fruits”). Finally, we foresee the possibility of creating a word embedding dataset for the Food Sciences, i.e., an analogue to GloVe embeddings trained on sensory-specific descriptions.

## Figures and Tables

**Table 1:** Results from the test set (BB/WJ combined) when using WA/WC combined as the training and validation data.

	Accuracy (%)	Precision	Recall	F1-Score
Parts of Speech	47.863	0.209	0.946	0.3422
LSTM	90.000	0.779	0.422	0.547

**Table 2:** Training results from combining the datasets and splitting train/test along tokens.

100% of Data Used	Loss	Accuracy (%)
Train	0.00238	99.9234
Validation	0.00153	99.946
Test	N/A	99.910
Parts of Speech	N/A	51.344

**Table 3:** Test results from combining the datasets and splitting train/test along tokens.

	Precision	Recall	F1-Score
Parts of Speech	0.47410	0.92496	0.62688
LSTM	0.99883	0.99912	0.99898

## Data Availability

The data presented in this study are available on request from the corresponding author. The data are not publicly available due to privacy concerns and copyright.

## Abbreviations

The following abbreviations are used in this manuscript:

DA	Descriptive Analysis
NLP	Natural Language Processing
LSTM	Long Short-Term Memory
WA	Whisky Advocate
WC	Whisky Cast
WJ	The Whiskey Jug
BB	Breaking Bourbon
CSV	Commab Separated Value
POS	Parts of speech

## References

[1] Buck L.B. (2004). Olfactory Receptors and Odor Coding in Mammals. Nutr. Rev..

[2] Heymann H., King E.S., Hopfer H., Varela P., Ares G. (2014). Classical Descriptive Analysis. Novel Techniques in Sensory Characterization and Consumer Profiling.

[3] Lawless L.J., Civille G.V. (2013). Developing Lexicons: A Review. J. Sens. Stud..

[4] Drake M., Civille G. (2003). Flavor Lexicons. Compr. Rev. Food Sci. Food Saf..

[5] Shapin S. (2016). A taste of science: Making the subjective objective in the California wine world. Soc. Stud. Sci..

[6] Varela P., Ares G. (2012). Sensory profiling, the blurred line between sensory and consumer science. A review of novel methods for product characterization. Food Res. Int..

[7] Ickes C.M., Lee S.Y., Cadwallader K.R. (2017). Novel Creation of a Rum Flavor Lexicon Through the Use of Web-Based Material. J. Food Sci..

[8] Valente C.C. (2016). Understanding South African Chenin Blanc Wine by Using Data Mining Techniques Applied to Published Sensory Data. Ph.D. Thesis.

[9] Wishart D., Kiers H.A.L., Rasson J.P., Groenen P.J.F., Schader M. (2000). Classification of Single Malt Whiskies. Data Analysis, Classification, and Related Methods.

[10] Bécue-Bertaut M., Pagès J. (2008). Multiple factor analysis and clustering of a mixture of quantitative, categorical and frequency data. Comput. Stat. Data Anal..

[11] Moroz D., Pecchioli B. (2019). Should You Invest in an Old Bottle of Whisky or in a Bottle of Old Whisky? A Hedonic Analysis of Vintage Single Malt Scotch Whisky Prices. J. Wine Econ..

[12] Hennion A. (2007). Those Things That Hold Us Together: Taste and Sociology. Cult. Sociol..

[13] Shapin S. (2012). The sciences of subjectivity. Soc. Stud. Sci..

[14] Lombardo C. (2018). Straight Up: Industry Revenue Will Steadily Grow as the Number of Independent Distillers Rises.

[15] McAuley J., Leskovec J., Jurafsky D. Learning Attitudes and Attributes from Multi-aspect Reviews. Proceedings of the 2012 IEEE 12th International Conference on Data Mining.

[16] Tao D., Yang P., Feng H. (2020). Utilization of text mining as a big data analysis tool for food science and nutrition. Compr. Rev. Food Sci. Food Saf..

[17] Hochreiter S., Schmidhuber J. (1997). Long short-term memory. Neural Comput..

[18] Ilin I., Chikin V., Solodskih K. (2018). Deep Learning for Specific Information Extraction from Unstructured Texts. https://towardsdatascience.com/deep-learning-for-specific-information-extraction-from-unstructured-texts-12c5b9dceada.

[19] Hamilton L.M., Lahne J. (2020). Fast and automated sensory analysis: Using natural language processing for descriptive lexicon development. Food Qual. Prefer..

[20] Ongaro L., White D., Sorel D. jQCloud. https://mistic100.github.io/jQCloud/.

[21] Pustejovsky J., Stubbs A. (2012). The Basics. Natural Language Annotation for Machine Learning.

[22] Symoneaux R., Galmarini M.V., Varela P., Ares G. (2014). Open-Ended Questions. Novel Techniques in Sensory Characterization and Consumer Profiling.

[23] Pennington J., Socher R., Manning C.D. GloVe: Global Vectors for Word Representation. Proceedings of the 2014 Conference on Empirical Methods in Natural Language Processing (EMNLP).

[24] Devlin J., Chang M., Lee K., Toutanova K. (2018). BERT: Pre-training of Deep Bidirectional Transformers for Language Understanding. arXiv.

[25] Comet.ML Home Page. https://www.comet.ml/.

[26] van der Maaten L.J.P., Hinton G.E. (2008). Visualizing High-Dimensional Data Using t-SNE. J. Mach. Learn. Res..

[27] Meyners M., Castura J.C., Varela P., Ares G. (2014). Check-All-That-Apply Questions. Novel Techniques in Sensory Characterization and Consumer Profiling.

[28] Greenacre M.J. (2017). Correspondence Analysis in Practice.

